# Effect of zinc versus vitamin A supplementation on pediatric patients with community-acquired pneumonia

**DOI:** 10.3389/fphar.2022.933998

**Published:** 2022-08-30

**Authors:** Aya Saied, Radwa Maher El Borolossy, Mourad Alfy Ramzy, Nagwa A. Sabri

**Affiliations:** ^1^ Clinical Pharmacist, Al Galaa Military Medical Complex, Cairo, Egypt; ^2^ Clinical Pharmacy Department, Faculty of Pharmacy, Ain Shams University, Cairo, Egypt; ^3^ Assistant Director of the Military Medical Services, Cairo, Egypt

**Keywords:** pneumonia, micronutrient, zinc, vitamin A, effusion, hospital stay, intensive care, pediatric

## Abstract

**Background:** Community-acquired pneumonia (CAP) is one of the most common infectious diseases affecting the respiratory tract and is responsible for a high mortality rate in children less than 5 years of age. The mortality rate due to CAP is much higher in low/middle-income countries than in high-income countries due to malnutrition and different micronutrient deficiencies that weaken the immune system.

**Aim:** The aim of this study was to compare the effects of zinc and vitamin A, as two elements of micronutrient agents, on the recovery rate of children suffering from CAP aged from 6 months to 5 years. The length of hospital stays was also investigated.

**Method:** A comparative, randomized, open-label, controlled, interventional study was carried out among children less than 5 years of age in the pediatric intensive care unit (PICU) diagnosed with CAP who were randomly divided into three groups. In addition to the standard therapy, group 1 was given zinc, group 2 was given vitamin A, and group 3 was the control group, given the standard therapy only. We compared the three groups in terms of recovery rate and hospital stay.

**Result:** The duration of hospitalization following zinc and vitamin A supplementation was reduced by an average of 3.21 days (95% CI: 5.01–1.41, *p* = 0.01) and 2.43 days (95% CI: 4.29–0.57, *p* = 0.01), respectively, compared to the control group. In addition, the two groups of vitamin A and zinc supplementation were associated with a shorter duration of pneumonic effusion (*p* < 0.001) in comparison to the control group. Additionally, there was no significant difference between the effects of zinc and vitamin A when compared to each other in terms of duration of hospital stay and pneumatic effusion.

**Conclusion:** The administration of zinc or vitamin A supplementation proved to be useful as an add-on therapy in community-acquired pneumonia, where it reduced the length of hospital stay and the duration of pneumonic effusion in pneumonic children less than 5 years of age.

## Introduction

Pneumonia is one of the infectious diseases that affect the lower respiratory tract system (Katz and Williams, 2018). Pneumonia is an acute infection affecting the lung and alveoli that can be caused by many different micro-organisms (viruses, bacteria, or fungi) (Li et al., 2020). in which pathogens enter the lung, causing inflammation and weakening the gas exchange mechanism between alveoli and pulmonary capillaries (Jafarzadeh and Oscuii, 2019; Hamed et al., 2019).

Community-acquired pneumonia (CAP) is the most common type of pneumonia where individuals are affected by the pathogen away from hospitals or other healthcare facilities (Symington and Scott, 2017). In addition, it is considered a leading cause of morbidity and mortality around the world, especially in children under 5 years of age (Hamed et al., 2019; Harel-Sterling et al., 2019).

Additionally, community-acquired pneumonia is defined as an acute infection of the pulmonary parenchyma associated with at least some symptoms of acute infection, accompanied by the presence of an acute infiltrate on a chest radiograph or auscultatory finding consistent with pneumonia (such as altered breath sound and/or localized roles), in neither hospitalized patient nor residing in a long-term care facility for more than 14 days before the onset of symptoms (Basnet et al., 2012; Baek et al., 2018).

It is worthy to mention that around the world, CAP is considered the major cause of death in children (Mahalanabis et al., 2002; Srinivasan et al., 2012). Also, every year, millions of children younger than 5 years died; moreover, the annual incidence of pneumonia in children younger than 5 years of age is 34–40 cases per 1000 in high-income countries. On the other hand, the incidence approaches 2 to 10 times higher in low and middle-income countries (LMICs) than in the high-income countries. Regarding the severity of infection and mortality rate, they are higher in LMICs (Srinivasan et al., 2012; Sakulchit, 2017).

One of the most common responsible co-factors for the high incidence of death due to CAP and LMICs is micronutrient deficiencies which have an important role in the regulation of essential defense mechanisms of the human body against infectious diseases and are very essential as well for appropriate cellular and molecular functions, including the cell of immunity system (Schulz et al., 2010; Kuti et al., 2021).

It is well known that childhood malnutrition affects both innate and adaptive immune function in pediatrics as well as impair the ability of the immunity system to produce an appropriate immune response against infections (Basnet et al., 2012; Walker and Black, 2012; Kuti et al., 2021).

Essential micronutrients, including zinc and vitamin A, have an important role in many vital pathways affecting immunity and human health, where, their deficiency is considered one of the risk factors for respiratory infections (Reyes et al., 2002; Hoang and Han, 2021).

Zinc is essential for gene expression and has an important role in nucleic acid and protein synthesis, cell replication, tissue growth and repair, and is vital for the regulation of immune system function and defense mechanisms against infectious micro-organisms (Morse and High, 2018; Wang and Song, 2018; Mumtaz et al., 2019; Hoang and Han, 2021; Jegalakshmi et al., 2022).

Moreover, it could inhibit angiotensin-converting enzyme 2 (ACE2) activity, loading to impair the interaction between the spike proteins of many micro-organisms and the host cells (Skalny et al., 2020; Hunter et al., 2021; Ateya, A.M. et al., 2021).

In addition, it reduces the permeability of the cell membrane and inhibits the transcapillary movement of plasma protein, which results in a consecutive decrease in inflammation and local edema (Hunter et al., 2021). On the other hand, zinc deficiency causes thymus atrophy, decreases T-helper cell and B cell production, and increases the susceptibility of micro-organisms to produce toxins (Skalny et al., 2020; Jalal et al., 2021).

Concerning, vitamin A, it is one of the most important micronutrients, which is essential for many vital functions such as cell growth, epithelial integrity, and immune system response, and is considered an anti-infectious vitamin due to its essential role in immune function by controlling the discrimination of T-helper cells to Th2 and production of interleukin-4 and interleukin-5, regulating the humoral antibody response, and inhibition of bacteria growth (Abd El-Shaheed et al., 2018; Shahzad et al., 2018). Vitamin A also acts as an antioxidant agent, preventing oxidative stress and cell damage (Khadim and Al-Fartusie, 2021).

It is well known that vitamin A is not synthesized in the human body and thus should come from the diet (Erdfelder et al., 1996; Imdad et al., 2017). According to the WHO, there are about 250 million children in LMICs who are suffering from vitamin A deficiency, resulting in a high rate of morbidity and mortality in children less than 5 years old (Abd El-Shaheed et al., 2018).

Many studies have been conducted about the effect of micronutrients in the treatment of CAP and its role in the acceleration of recovery and the decrease of complications and mortality rates in children (Reyes et al., 2002; West, 2003; Shakur et al., 2009; Valavi et al., 2011; Shah et al., 2012; Imdad et al., 2017; Morse and High, 2018; Saleh and Abo El Fotoh, 2018; Wang and Song, 2018; Hoang and Han, 2021; R Prasad et al., 2019). According to our knowledge, there is no comparative study evaluating the effect of zinc versus vitamin A in pediatric pneumonia.

Thus, the aim of our study was to investigate the efficacy and safety of zinc supplementation compared to vitamin A as an adjuvant to the standard protocol of therapy used in the management of pneumonia in pediatric patients. This was performed through the evaluation of the role of each on the recovery rate in pediatric patients with pneumonia in terms of hospital stay and pneumonic effusion duration.

This study was made by collaboration between the physicians of the pediatric intensive care unit and a clinical pharmacist. Physicians diagnose the patient's clinical situation, prescribe the medication, and monitor the vital signs of the patient. While the clinical pharmacist's concern is the optimized use of medication, by making sure that every patient takes his right and optimal medication according to his clinical situation, check the dose and administration, and being aware of the side effect of each drug to decrease the medication error and achieve the optimize use of medication (Hepler, 2004; Alagha et al., 2011).

## Patients and methods

### Design

A comparative, randomized, open-label, controlled, pilot, and interventional study was carried out from January 2019 until February 2020. All cases included in the study were patients in the intensive care unit due to severe pneumonia. Patients included in the study were randomly divided into three groups with 25 cases in each one.

### Setting

Pediatric intensive care unit (PICU) of the pediatric and gynecology specialized hospital at Al Galaa Military Complex and Ghamra military hospital, Cairo, Egypt.

### Inclusion criteria

- Patients admitted to the pediatric intensive care unit were diagnosed with community-acquired pneumonia
- Age from 1 to 60 months
- Patient’s health status, which allowed oral medication

### Exclusion criteria

Children with known heart disease, children on medication with zinc supplements or vitamin A, children with obstructive air-way disease or known case of asthma, children NPO (nothing by mouth) on a ventilator, children with active measles and known intolerance or allergy to zinc/vitamin A or zinc/vitamin A-containing products.

### Method

#### Sample size calculation

The sample size was calculated by GPower v.3.1.9.4 (Erdfelder, E., Faul, F., and Bates, 1995). According to Lynne M. Connelly (2008), extant literature suggests that a pilot study sample should be around 10% of the sample project for the larger parent study.

Therefore, for a full-scale study, where the main dependent variables are hospital stay and post-treatment laboratory parameters. Using multiple linear regression with seven predictors (baseline value, age, gender, ABW, pneumonia grade, effusion presence, and treatment), expecting a modest effect on hospital stay and laboratory parameters a priori (effect size/Cohen’s *f* ^2^ = 0.02), with the desired power of 95% to reject the null hypothesis (H_o_: no increase in R^2^ by adding the grouping/treatment variable), and a type-I error probability not more than 0.05. For our pilot study, the required total sample size was decided to be 75 participants, equally allocated among the 3 groups (25 participants in each group).

A total of 75 eligible patients were divided into three groups (Supplementary Figure S1):

• Group (1) (zinc group): patients were administered zinc (Zinc Origin®, zinc sulfate syrup 10mg/5ml, manufacturing by Origin International Pharms, Egypt), in addition to the standard therapy, given for 7 days as follows; 10 mg once daily for children aged less than 1 year, or 20 mg once daily for children aged over 1 year to 5 years
• Group (2) (vitamin A group): in addition to the standard therapy for pneumonia, patients were given vitamin A (A-VITON®, vitamin A 50,000 IU soft gelatin capsule, manufactured by Kahera Pharma-Egypt) for 5 days on the dose of 50,000 international unit (IU) at day one and day five for children aged less than 1 year, or 100,000 international unit (IU) at day one and day five for children aged over 1 year to 5 years
• Group (3) (control group): patients were given the standard therapy and medication for the treatment of pneumonia

The standard therapy consisted of the following medications: empirical antibiotic treatment including vancomycin 15 mg/kg/8 h, meropenem 20 mg/kg/8 h, colistin 25000 IU/kg/8 h, and if suspected anaerobic infection they were given imipenem/cilastatin 20 mg/kg/6 h. The empirical antibiotics were exchanged after culture to sensitive antibiotics. Additionally, empirical antifungal treatment was given in case of suspected infection or for patients who stayed for more than 6 days in the ICU. Fluconazole 6 mg/kg/24h, antipyretic; paracetamol 15 mg/kg/dose up to 6 times daily, inhaler; ipratropium/salbutamol-albuterol, budesonide, saline, and anti-inflammatory agent; dexamethasone 0.15 mg/kg/6 h.

#### Clinical assessment and laboratory evaluation

1) Physical examination and vital sign assessment: the physician underwent physical investigations daily and nurses assessed body temperature, respiratory rate, and oxygen saturation was monitored daily through pulse oximetry
2) Laboratory data evaluation: on admission and every 3 days assessment of C-reactive protein, arterial blood gases [PCO2, PO2, and pH], kidney function [serum creatinine and blood urea nitrogen (BUN)], and liver function alanine aminotransferase (ALT), aspartate aminotransferase (AST) were performed
3) Monitoring of the expected side effects of the interventional medications: the side effects of zinc supplementation include nausea, vomiting, diarrhea, tarry stool, and stomach pain, while those of vitamin A include nausea, vomiting, diarrhea, liver damage, blurry vision, and skin irritation were monitored daily through a side effect monitoring form, where the appearance of any of the previously mentioned side effects were reported and the method of handling it as well was reported

### Outcomes

• Primary outcome: the role of zinc supplementation and vitamin A on the recovery rate in the pediatric patient with pneumonia in terms of hospital stay and pneumonic effusion duration.
• Secondary outcome: evaluation of the safety of zinc supplementation and vitamin A in pediatric patients with pneumonia in terms of proportion of adverse drug events and complications.

### Ethical consideration

The study protocol was revised and approved (under no; 228) by the institutional review board of the ethics committee of faculty of pharmacy, Ain Shams University which is registered at the Egyptian Ministry of Health (MOH).

The current study was conducted according to the code of ethics of declaration (Hunter et al., 2021a; Hunter et al., 2021) (Review et al., 2014), communication and principles, and followed the CONSORT guidelines (Schulz et al., 2010; Ateya et al., 2021).

The caregivers of the eligible children were informed about the study and asked to sign a written informed consent before starting the study without any obligation on them to continue if they did not want to.

### Statistical analysis

The statistical analysis was performed using R software version 3.6.1. Two-sided *p*-values less than 0.05 were considered statistically significant. The normality assumption was tested by Shapiro–Wilk’s test; normally distributed continuous data were represented by mean and standard deviation and were compared using Student’s t-test or one-way analysis of variance (ANOVA); skewed data were represented by median and range and were compared using the Mann–Whitney’s test or Kruskal–Wallis test. Tukey’s HSD (honestly significant difference) test was performed after significant ANOVA.

Age was categorized as infants (birth to ≤12 months), toddlers (>12 months to ≤36 months), and preschools (>36 months). Subjects were categorized according to their weight-for-age or weight-for-height to underweight, healthy, overweight, and obese using the CDC and WHO growth charts.

Multiple linear regression analysis was performed to quantify the marginal effect of treatment with vitamin A or zinc (vs. control) on the duration of hospitalization while controlling for age, gender, bodyweight category, pneumonia grade, and presence of effusion. Two-way repeated-measures ANOVAs were also performed to quantify the effect of treatment with vitamin A or zinc on post-treatment vital signs and sepsis-related lab parameters, including temperature, respiratory rate, O_2_ saturation, hemoglobin level, total leukocyte count, platelet count, CRP, ALT, and serum creatinine.

## Results

### Patients’ demographics

A total of 75 patients completed the study without dropouts. The median age of subjects was 30 months (range: 6–55 months). At baseline, the values of age, gender, actual body weight, and the weight/BMI-to-age or BMI-to-height CDC/WHO weight category, pneumonia grade, and presence/absence of pneumonic effusion were all comparable. Subjects’ baseline demographic characteristics are shown in Table 1.

**TABLE 1:** Baseline demographic and clinical characteristics of study participants.

Parameter	Control (*n* = 25)	Vitamin A (*n* = 25)	Zinc (*n* = 25)	Total (*n* = 75)	*p* value
Age (months)					0.26^a^
Median (range)	34.0 (6.0–55.0)	29.0 (6.0–52.0)	27.0 (6.0–54.0)	30.0 (6.0–55.0)	
Infant	2 (8.0%)	8 (32.0%)	2 (8.0%)	12 (16.0%)	
Toddler	11 (44.0%)	8 (32.0%)	14 (56.0%)	33 (44.0%)	
Preschool	12 (48.0%)	9 (36.0%)	9 (36.0%)	30 (40.0%)	
Gender					0.42^b^
Female, N (%)	13 (52.0%)	17 (68.0%)	13 (52.0%)	43 (57.3%)	
Male, N (%)	12 (48.0%)	8 (32.0%)	12 (48.0%)	32 (42.7%)	
ABW (kg)					0.14^c^
Mean (S.D)	13.5 (3.1)	11.8 (3.7)	11.8 (3.3)	12.3 (3.5)	
Underweight, N (%)	2 (8.0%)	3 (12.0%)	2 (8.0%)	7 (9.3%)	
Healthy, N (%)	10 (40.0%)	11 (44.0%)	11 (44.0%)	32 (42.7%)	
Overweight, N (%)	2 (8.0%)	3 (12.0%)	5 (20.0%)	10 (13.3%)	
Obese, N (%)	11 (44.0%)	8 (32.0%)	7 (28.0%)	26 (34.7%)	
Pneumonia grade					1.00^b^
Grade 3, N (%)	11 (44.0%)	11 (44.0%)	11 (44.0%)	33 (44.0%)	
Grade 4, N (%)	14 (56.0%)	14 (56.0%)	14 (56.0%)	42 (56.0%)	
Effusion					1.00^b^
Absent, N (%)	19 (76.0%)	19 (76.0%)	19 (76.0%)	57 (76.0%)	
Present, N (%)	6 (24.0%)	6 (24.0%)	6 (24.0%)	18 (24.0%)	

a Kruskal–Wallis rank-sum test.

b Pearson’s Chi-squared test.

c Analysis of variance linear model (ANOVA).

S.D: standard deviation, CDC: Center of Disease Control and Prevention, WHO: World Health Organization, BMI: body mass index, and ABW: actual body weight.

### Laboratory evaluation and physical examinations

At baseline, there was no significant difference in the patients’ clinical laboratory parameters and vital signs among the studied groups; additionally, no participant was presented with markers of organ damage (Table 2).

**TABLE 2:** Values of the patients’ laboratory parameters and vitals at baseline and at the end of the study.

	Baseline				After 5–7 days of treatment				At discharge			
	Control	Vitamin A	Zinc	*p* value	Control	Vitamin A	Zinc	*p* value	Control	Vitamin A	Zinc	*p* value
ALT (U/L)	36.2 (6.52)	35.7 (5.99)	37.1 (7.85)	0.76	35.7 (6.68)	35.0 (6.69)	35 (7.12)	0.93	34.4 (4.96)	33.7 (5.22)	33.8 (6.16)	0.88
AST (U/L)	38.6 (4.86)	38.4 (6.27)	39.4 (8.35)	0.85	37.4 (6.05)	37.2 (5.62)	36.2 (5.66)	0.74	34.2 (5.70)	35.2 (3.81)	35.2 (4.78)	0.71
BUN (mg/dl)	9.68 (1.55)	9.60 (1.08)	9.56 (1.42)	0.95	9.56 (1.04)	9.52 (0.82)	9.04 (0.98)	0.11	9.44 (0.82)	9.2 (1.00)	9.24 (0.88)	0.61
CRP (mg/L)	64.4 (59.9)	61.7 (42.9)	71.5 (54.0)	0.79	36.8 (37.4)	33.5 (33.2)	23.4 (20.9)	0.29	7.83 (2.34)	7.21 (2.64)	8.9 (4.41)	0.19
Hb (g/dl)	11.9 (1.35)	11.6 (1.34)	11.5 (1.73)	0.48	11.1 (1.14)	11.0 (1.45)	11.2 (1.5)	0.96	11.6 (0.92)	11.3 (1.1)	10.9 (2.44)	0.35
PCO_2_ (mmHg)	50.0 (17.3)	52.4 (16.0)	50.2 (17.9)	0.86	46.6 (8.75)	43.9 (8.05)	41.9 (6.49)	0.11	39.0 (1.9)	38.5 (2.47)	39.3 (3.95)	0.61
PH	7.27 (0.18)	7.18 (0.19)	7.23 (0.18)	0.22	7.35 (0.07)	7.35 (0.06)	7.37 (0.07)	0.40	7.40 (0.01)	8.61 (6.00)	7.40 (0.01)	0.37
Plt (×10^6^/L)	289 (68.4)	310 (73.7)	265 (98.4)	0.16	269 (76.2)	284 (71.6)	249 (97.7)	0.33	285 (85.1)	256 (76.4)	251 (111)	0.36
PO_2_ (mmHg)	59.5 (44.0)	45.2 (12.3)	55.8 (35.6)	0.30	75.1 (13.7)	78.2 (16.9)	85.6 (15.7)	0.05	96.0 (4.33)	95.1 (7.54)	95.7 (3.82)	0.86
RR (BPM)	47.7 (9.62)	47.2 (10.4)	48.8 (7.23)	0.82	37.6 (8.94)	36.8 (9.13)	34.5 (6.37)	0.40	34.4 (8.28)	32.7 (6.99)	32.4 (5.69)	0.55
SO_2_ (%)	80.5 (8.81)	79.3 (8.62)	80.8 (9.39)	0.82	94.4 (4.96)	93.4 (7.37)	97.1 (2.60)	0.32	98.4 (1.12)	94.8 (17.5)	98.5 (1.48)	0.34
SCr (mg/dl)	0.46 (0.11)	0.46 (0.10)	0.46 (0.10)	0.95	0.44 (0.06)	0.46 (0.08)	0.428 (0.08)	0.32	0.43 (0.05)	0.428 (0.05)	0.42 (0.08)	0.87
T (°C)	38.4 (0.88)	37.9 (0.95)	37.9 (0.78)	0.08	37.1 (0.15)	35.9 (6.00)	37.1 (0.16)	0.36	37.0 (0.10)	35.8 (6.02)	35.5 (5.88)	0.50
TLC (×10^6^/L)	22.4 (7.24)	22.5 (8.02)	40.2 (93.1)	0.41	15.7 (7.06)	14.0 (7.53)	27.5 (72.7)	0.47	8.44 (1.63)	7.52 (1.68)	18.9 (54.6)	0.37

*Statistical test used was one-way analysis of variance (ANOVA).

After intervention and at discharge, there was no significant difference among the studied groups concerning the clinical laboratory parameters and vital signs (Table 2). Moreover, no side effects were reported during the study due to the administration of zinc and vitamin A supplementation.

### Effect of zinc and vitamin A supplementation on the effusion’s days

Statistical analysis of the obtained results showed that both groups of vitamin A and zinc were associated with a significantly shorter duration of pneumonic effusion (*p*-value < 0.001) compared to the control group (Table 3).

**TABLE 3:** The duration of pneumonia effusion among the studied groups.

Term	Df	SSE	MSE	F-statistic	*p* value
Treatment	2	129	64.4	16.6	<0.001
Residuals	16	62.3	3.89		

Df: degrees of freedom, MSE: mean squared errors, SSE: sum of squared errors.

Post hoc contrasts showed that the both vitamin A and zinc groups were superior to the control one (Table 4). Duration of effusion was 3 days lower with zinc therapy compared to vitamin A. However, the difference between the effect of zinc and vitamin A was statistically insignificant (*p* value = 0.06).

**TABLE 4:** The post hoc contrast of the pneumonic effusion in the studied groups.

Contrast				95% CI		
	x̅_1_	x̅_2_	x̅_1_- x̅_2_	LB	UB	*p* value^a^
Vitamin A-control	8.83 (±0.75)	12.3 (±2.75)	−3.45	−6.28	−0.62	0.02
Zinc-control	6.00 (±1.67)	12.3 (±2.75)	−6.29	−9.12	−3.45	<0.01
Zinc-vitamin A	6.00 (±1.67)	8.83 (±0.75)	−2.83	−5.77	0.11	0.06

Tukey’s HSD test; CI, confidence interval; LB, lower bound; UB, upper bound.

### Duration of hospitalization

A follow-up two-way ANOVA (Tables 5, 6) showed a non-significant interaction between either pneumonia grade, presence/absence of effusion, and vitamin A/zinc (indicating independence of response to supplementation to pneumonia grade or effusion) (Supplementary Figures S2, S3).

**TABLE 5:** The duration of hospitalization regarding effusion presence or absence among the studied groups.

Term	Df	SSE	MSE	F-statistic	*p* value
Effusion	1	560	560	49.3	<0.001
Treatment	2	149	74.3	6.54	<0.01
Effusion-treatment	2	3.52	1.76	0.16	0.86
Residuals	69	784	11.4		

One-way analysis of variance (ANOVA) Df, degrees of freedom; MSE, mean squared errors; SSE, sum of squared errors.

**TABLE 6:** The duration of hospitalization regarding pneumonia grade among the studied groups.

Term	Df	SSE	MSE	F-statistic	*p* value
Grade	1	497	497	40.4	<0.001
Treatment	2	149	74.3	6.03	<0.01
Grade-treatment	2	0.35	0.18	0.01	0.97
Residuals	69	850	12.3		

One-way analysis of variance (ANOVA), Df: degrees of freedom, MSE: mean squared errors, and SSE: sum of squared errors.

Post hoc contrasts (Tables 7, 8) showed that both vitamin A and zinc supplementation were correlated with a significantly shorter duration of hospitalization in days compared to the control group.

**TABLE 7:** Tukey’s honestly significant difference post hoc contrasts.

Contrast	Mean difference^*^	95% CI		*p* value
		LB	UB	
Effusion				
Present—absent	−6.40	−8.19	−4.60	<0.001
Treatment				
Control—vitamin A	2.56	0.31	4.81	0.02
Control—zinc	3.28	1.03	5.53	<0.01
Vitamin A—zinc	0.72	−1.54	2.97	0.73

Based on marginal means. CI, confidence interval; LB, lower bound; UB, upper bound.

**TABLE 8:** Tukey’s honestly significant difference post hoc contrasts.

Contrast	Mean difference^*^	95% CI		*p* value
		LB	UB	
Pneumonia grade				
Grade 3–grade 4	−5.19	−6.79	−3.58	<0.001
Treatment				
Control—vitamin A	2.56	0.22	4.90	0.03
Control—zinc	3.28	0.94	5.62	<0.01
Vitamin A—zinc	0.72	−1.62	3.06	0.74

*Based on marginal means. CI, confidence interval; LB, lower bound; UB, upper bound.

In the multiple linear regression analysis (Table 9), vitamin A supplementation marginally reduced the duration of hospitalization by an average of 2.43 days (95% CI: 4.29**–−**0.57, *p*-value = 0.01). Similarly, zinc supplementation reduced the duration of hospitalization by an average of 3.21 days (95% CI: 5.01**–−**1.41, *p*-value < 0.01). Age, gender, and weight had no significant impact on the duration of hospitalization.

**TABLE 9:** Impact of vitamin A/zinc supplementation while controlling for demographics and patients’ factors.

Predictors	Estimates	95% CI	*p* value
Age category			
Infant	References		
Toddler	−0.01	−2.72–2.71	1.00
Preschool	1.06	−1.81–3.93	0.46
Gender			
Female	References		
Male	0.27	−1.25–1.78	0.73
Weight CDC/WHO category			
Healthy	References		
Obese	0.08	−1.65–1.81	0.93
Overweight	0.57	−1.78–2.92	0.63
Underweight	0.66	−2.38–3.71	0.67
Pneumonia grade			
Grade 3	References		
Grade 4	3.16	1.41–4.90	<0.01
Effusion			
Absent	References		
Present	3.89	1.68–6.11	<0.01
Treatment			
Control	References		
Vitamin A	−2.43	−4.29–-0.57	0.01
Zinc	−3.21	−5.01–-1.41	<0.01

R2/R2 adjusted 0.586/0.521. CI, confidence interval; CDC, Center of Disease Control and Prevention; WHO, World Health Organization.

## Discussion

Zinc and vitamin A are micronutrients that have biological activities related to the immune system and recovery after lower respiratory tract infections, and their concentration is usually reported as lower than required in children of LMICs (Gupta et al., 2020; Afolami, 2021).

Zinc has an essential role in the metabolisms of cells and immunity. Its deficiency causes a defect in the integration of epithelial cells of the lung (Shakur et al., 2009). It is well known that zinc is effective in the treatment of respiratory tract infection (RTI) as it reduces the duration of symptoms as well as decreases the severity of the disease, (Hunter et al., 2021), through its ability to inhibit an angiotensin-converting enzyme 2 (ACE2) and consequently impairs the ability of infection (McPherson et al., 2020) ***.*** In addition, zinc was found to reduce the percentage of developing mild to moderate symptoms consistent with RTI as it decreases the respiratory rate, body temperature, and influenza-like illnesses (Hunter et al., 2021).

On the other hand, vitamin A is considered one of the broadly studied micronutrients regarding its effect on the immune system **(** Karter et al., 1995) and is named anti-infectious vitamin owing to its effect on the regulation of human immune function **(**Bates et al., 1995). It was reported by Zhang et al., and West et al., that vitamin A deficiency causes recurrent respiratory tract infection as it is one of the fat-soluble vitamins which have a great effect on the health of the immunity system while its deficiency for a long time increases the morbidity and mortality level, especially in the pediatric population (Zhang et al., 2019).

In a cross-sectional study performed by Abd El-Shaheed et al. (2018) reported that there was a positive association between vitamin A and both CD4 and Tβ4, thus, vitamin A deficiency reduced the efficacy of the immunity system against infectious diseases. In another clinical trial, it was reported that vitamin A intervention caused a decrease in the duration of fever and hospital stay **(** Li et al., 2020a). In terms of hospital stay, we found that the interventions with zinc or vitamin A reduced days of hospitalization by an average of 3.21 days (95% CI: 5.01–−1.41, *p* < 0.01) and 2.43 days (95% CI: 4.29–−0.57, *p* = 0.01), respectively. This result is similar to those found in the study by Valavi et al. (2011), where they used zinc at 2 mg/kg/day (max. daily dose: 20 mg) for 5 days for children from 3 to 60 months old, and those reported in the study by Reyes et al. (2002), who used zinc at 15 mg twice daily until discharge from hospital or up to maximum 7 days for children from 2 to 60 months old.

Concerning the significance of the reduction in length of hospital stay in the current study, it is important to mention that reduction in the length of hospital stay is an indicator of good medical practice and the efficacy of the treatment protocol (Baek H. et al., 2018). Moreover, there is a criterion for hospital discharge depending on the hospitalized case, according to Marrie et al., a patient with pneumonia is considered ready for discharge when his vital signs have been normalized, lung function and oxygenation have returned to the baseline status, results of blood culture is negative, and the blood cell count is lower than 12×10^9^/L (Marrie et al., 2000; Moeller et al., 2006).

On the other hand, Nacul et al. (1998) reported that the results obtained in their study required more evidence to recognize the effect of vitamin A on the recovery of pneumonia by using two different doses of vitamin A (100000–200000 IU per dose) for children from 1 to 48 months old. However, Shah et al. (2012) found that zinc had no significant reduction effect on hospital stay in pneumonia patients upon the administration of 10 mg twice daily for 7 days for children from 2 to 60 months old.

In the present study, although the zinc group showed a much lower length of stay than vitamin A, on comparing the effects of zinc and vitamin A on hospitalization length of stay, the difference was not statistically significant which might be attributed to the short period of the study due to the short total period of patients’ hospital residence either in the ICU or the inpatient ward, according to the physicians’ decisions, not the researchers, where, this obtained difference might be significant in case of a longer period of supplementation administration time.

Pleural effusion is a pathological condition where excess fluid accumulates between the visceral and parietal pleural membranes that surround the lungs. This condition causes impaired lung function due to the fluid between the tissues in the pleural cavity (Arad et al., 2009; Bai et al., 2019). In the present work, the duration of effusion in the groups of zinc and vitamin A was found to be significantly lower compared to the control group. On comparing the zinc group to the vitamin A group, it was found that zinc causes a 3 days non-significant reduction in the duration of effusion compared to vitamin A.

The mechanisms by which zinc and vitamin A cause the reduction in the duration of hospital stay or pleural effusion are unknown, but it could be due to their antibacterial and anti-inflammatory properties, as well as their role in tissue growth regulation (Shahzad et al., 2018; Skalny et al., 2020; Khadim and Al-Fartusie, 2021). Zinc could be more effective than vitamin A due to zinc’s activities in reducing the susceptibility of micro-organisms to producing toxins and its role in regulating the cell membrane permeability of water and minerals (Skalny et al., 2020; Hunter et al., 2021).

At the end of the present study period, a significant difference regarding the clinical assessment and laboratory values found among the studied groups similar to that reported in the study by Srinivasan et al. (2012), Valentiner- Branth et al. (2010) regarding zinc supplementation, and Nacul et al. (1998) for vitamin A supplementation.

In the study by Shah et al. (2012) and Valentiner- Branth et al. (2010); it was found that the female patients had lower susceptibility to pneumonia infection than the male patients, where the recorded percentages were 35.35% and 42%, respectively, but in our study, we found that the female patients had higher susceptibility (57.3%) which may be related to a general condition where the mean of hemoglobin and total leukocyte count of female participated in our study at admission were 11.5 g/dl and 33.01 × 10^9^/L, respectively, which was lower than the mean in male participants, 11.7 g/dl and 22.1 × 10^9^/L, respectively.

## Conclusion

Administration of either zinc or vitamin A supplementation in community-acquired pneumonia in children aged less than 5 years significantly reduces the hospitalization stay and duration of effusion compared to the control group with a non-significant difference between the effect of zinc and/or vitamin A supplementation.

Based on our knowledge, this is the first clinical study that assesses and compares the effect of zinc and vitamin A as supplement treatment in community-acquired pneumonia in children less than 5 years old in terms of hospital stay and pleural effusion. So, we hope that our funding could be helpful for more clinical trials that may use our results to test the significance between the effect of zinc and vitamin A by using different duration of administration or assessing other parameters.

## Limitations

This is a preliminary study to investigate the effect of zinc versus vitamin A in pediatric patients with community-acquired pneumonia performed on a limited number of patients being a preliminary (pilot) study; the relatively small sample size of our study may reduce the ability to determine the statistical significance of the variables. It is recommended that further large-scale studies be performed on a large number of patients. A second limitation was the measurement of micronutrient concentration, as both zinc and vitamin A levels, which were not detected at baseline.

## Data Availability

The original contributions presented in the study are included in the article/Supplementary Material; further inquiries can be directed to the corresponding author.
