# Supplementary figures and images for: Effect of zinc versus vitamin A supplementation on pediatric patients with community-acquired pneumonia
Source: Front Pharmacol. 2022 Aug 30;13:933998. doi: 10.3389/fphar.2022.933998 (PMC9468708; doi:10.3389/fphar.2022.933998)

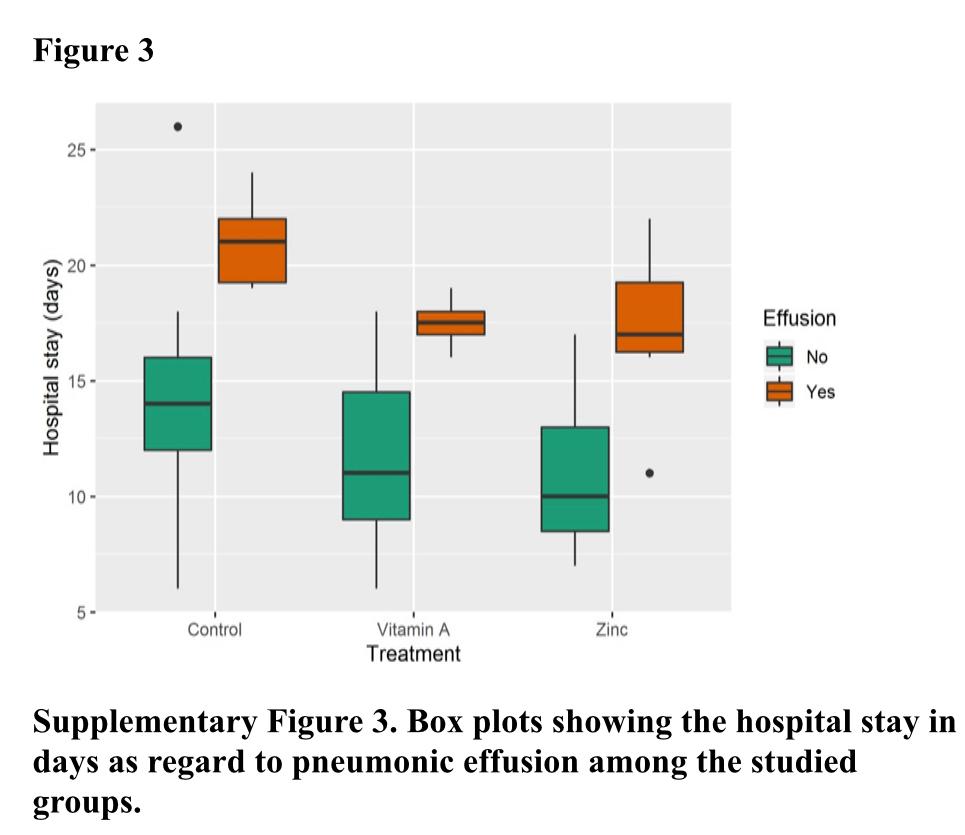

Supplement: Supplementary file 1 [file Image3.jpeg]

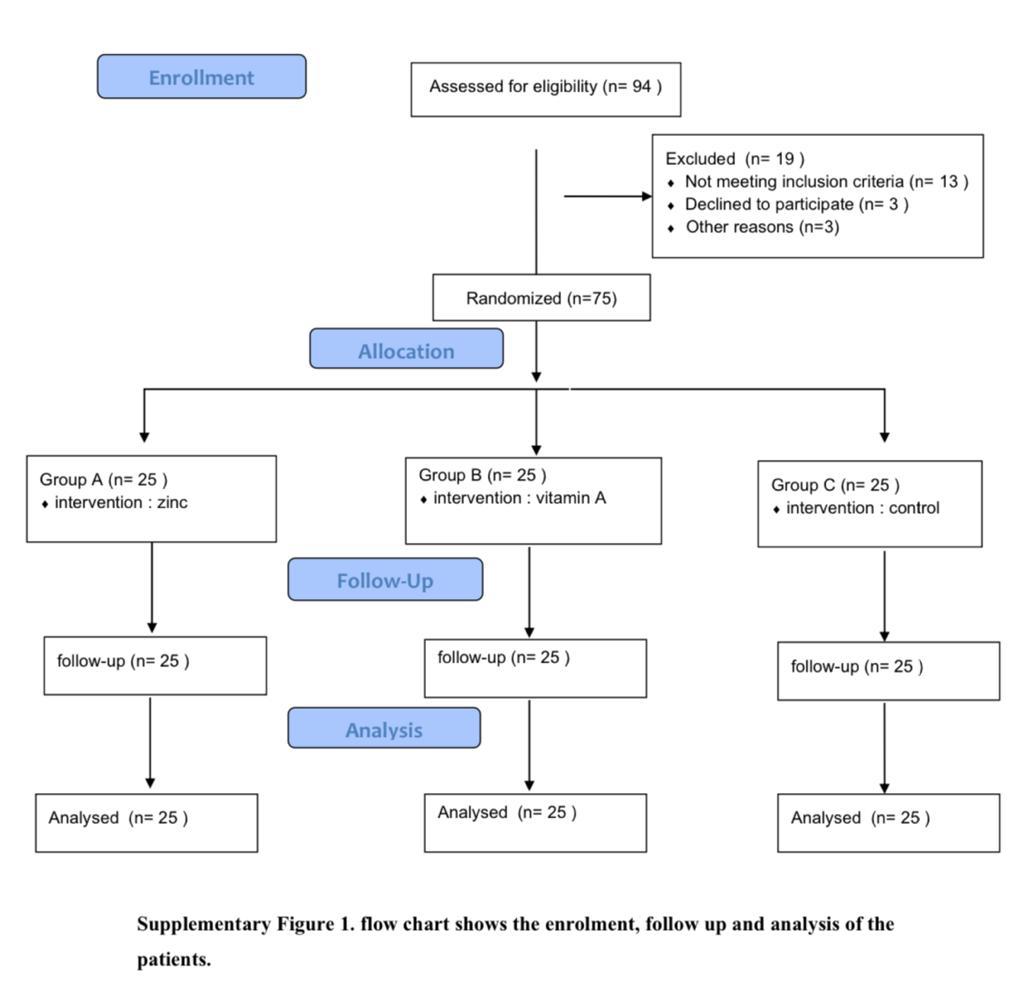

Supplement: Supplementary file 2 [file Image1.jpeg]

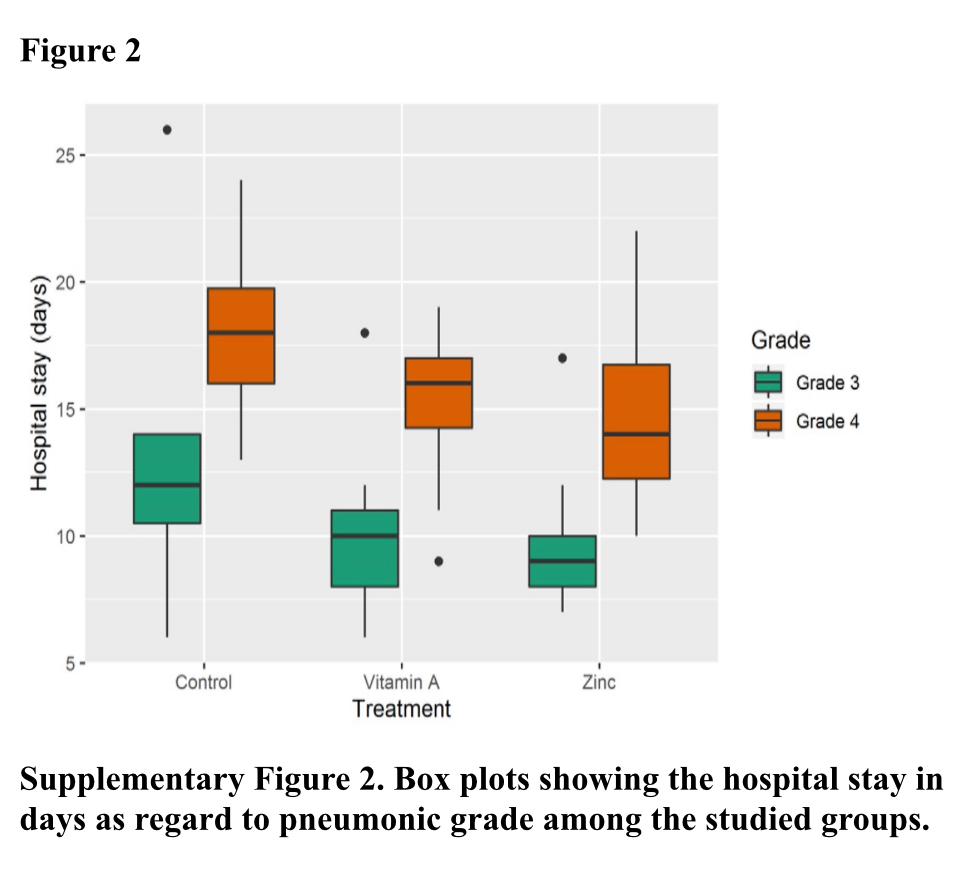

Supplement: Supplementary file 3 [file Image2.jpeg]
